# Non-Mineral Antioxidant Supplementation in Endometriosis: Biological Rationale, Clinical Evidence, and Therapeutic Implications—A Narrative Review

**DOI:** 10.3390/nu18081182

**Published:** 2026-04-09

**Authors:** Kamila Pokorska-Niewiada, Katarzyna Janda-Milczarek, Khasan Kayumov, Maciej Ziętek, Małgorzata Szczuko

**Affiliations:** 1 Department of Toxicology, Dairy Technology and Food Storage, West Pomeranian University of Technology in Szczecin, 71-454 Szczecin, Poland; 2 Department of Biology, Parasitology and Pharmaceutical Botany, Pomeranian Medical University in Szczecin, 70-111 Szczecin, Poland; katarzyna.janda.milczarek@pum.edu.pl; 3 Department of Human and Animals Physiology, National University of Uzbekistan named after Mirzo Ulugbek, Tashkent 100174, Uzbekistan; qayumovhasan642@gmail.com; 4 Department of General Pharmacology and Pharmacoeconomics, Pomeranian Medical University in Szczecin, 71-460 Szczecin, Poland; maciej.zietek@pum.edu.pl; 5 Department of Bromatology and Diagnostic Nutrition, Pomeranian Medical University, 70-111 Szczecin, Poland

**Keywords:** endometriosis, oxidative stress, non-mineral antioxidants, melatonin, *N*-acetylcysteine, coenzyme Q10, vitamins

## Abstract

**Background/Objectives**: Oxidative stress plays an important role in the pathophysiology of endometriosis, contributing to inflammation, immune dysregulation, and lesion progression. This has led to growing interest in antioxidant-based strategies as potential supportive interventions. **Methods**: A literature search was conducted using PubMed, Scopus, and Web of Science databases, covering studies published from database inception until the end of January 2026. The review focused on clinically relevant endpoints, including pain intensity, markers of inflammation and oxidative stress, reproductive parameters, and quality of life. **Results**: Among the analyzed interventions, the most consistent clinical effects were observed with melatonin, with randomized controlled trials indicating a moderate reduction in pain. *N*-acetylcysteine shows potentially beneficial effects; however, the available clinical data remain limited and heterogeneous. For other supplements, the evidence is inconsistent or insufficient to support clear clinical conclusions, and in many cases relies on indirect or mechanistic findings rather than well-established clinical outcomes. **Conclusions**: Current evidence does not support the use of non-mineral antioxidant supplements as standalone therapy for endometriosis. They may be considered as adjunctive strategies, although their clinical effectiveness remains uncertain and requires confirmation in well-designed randomized clinical trials.

## 1. Introduction

Endometriosis is a chronic gynecological disease characterized by the presence of endometrium-like tissue outside the uterine cavity and persistent estrogen-dependent inflammation. The prevalence of the disease ranges from 10 to 15% among women of reproductive age, and it is recognized as a primary contributor to chronic pelvic pain and infertility [1,2]. Despite its high prevalence, the diagnosis of endometriosis is often significantly delayed, and the available treatment methods are mostly symptomatic, with limited long-term effectiveness and a risk of side effects [3,4]. The pathophysiology of endometriosis is intricate and involves the interaction of hormonal disorders, immune system dysfunction, chronic inflammation, and abnormal angiogenesis [5,6,7]. In recent years, mounting attention has been directed toward oxidative stress as a mechanism that fosters the perpetuation of inflammatory processes and disease progression. In this context, nutritional factors and antioxidant-based interventions that may influence redox homeostasis and modulate endometriosis-related inflammatory processes are of interest. Observational studies suggest a potential association between a diet rich in antioxidants and milder symptoms; however, the results of interventional studies on supplementation remain inconclusive [8,9,10].

Considering the proliferation of publications and the heterogeneity of study designs and endpoints evaluated, a critical synthesis of the available data is imperative. The objective of this review is to evaluate the current evidence on the role of non-mineral antioxidants in endometriosis, with particular emphasis on their potential impact on clinically relevant symptoms such as pain severity, fertility, and quality of life.

Given the breadth of the topic and the heterogeneity of the available literature, the present review focuses on selected non-mineral antioxidants chosen based on a combination of factors, including biological relevance to oxidative stress-related mechanisms, availability of clinical data, and frequency of investigation in the literature. This approach was intended to maintain clinical relevance rather than to provide an exhaustive overview of all redox-modulating compounds. Due to substantial variability in study design, patient populations, interventions, and outcome measures, the findings are presented as a qualitative synthesis and should be interpreted with caution. Other redox-modulating compounds were not included, as they fall outside the defined scope of this review.

The novelty of this review lies in combining mechanistic insights into oxidative stress with a critical evaluation of clinical evidence, with particular attention to the discrepancy between biological plausibility and the limited clinical effectiveness of antioxidant supplementation in endometriosis.

## 2. Materials and Methods

### 2.1. Review Design

This review was conducted as a structured narrative review based on a systematic search of the literature, with particular attention to clinical studies evaluating non-mineral antioxidant supplementation in endometriosis. In addition to clinical evidence, experimental and mechanistic studies related to oxidative stress pathways were considered to provide biological context for the observed effects. Mechanistic and experimental findings were interpreted separately from clinical evidence and should not be considered directly translatable into clinical outcomes.

Because the available studies differed substantially regarding study design, intervention type, treatment duration, outcome measures, and patient populations, a quantitative meta-analysis was not performed. The findings were therefore synthesized narratively, with attention to the clinical relevance and methodological limitations of the included studies.

### 2.2. Literature Search Strategy

A literature search was performed in PubMed/MEDLINE, Scopus, and the Web of Science to identify publications relevant to oxidative stress and non-mineral antioxidant supplementation in endometriosis. The search covered studies published from database inception until the end of January 2026.

Search terms included combinations of keywords such as “endometriosis”, “oxidative stress”, “antioxidants”, “vitamin C”, “vitamin E”, “vitamin D”, “melatonin”, “*N*-acetylcysteine”, and “Coenzyme Q10”. Boolean operators (AND, OR) were used to refine the search strategy.

No restrictions were applied regarding study design at this stage. The search was limited to articles published in English.

### 2.3. Eligibility Criteria

Studies identified through the search strategy were screened according to predefined inclusion and exclusion criteria. Studies evaluating non-mineral antioxidant supplementation in endometriosis and reporting clinically relevant outcomes, such as pain intensity, oxidative stress markers, inflammatory parameters, reproductive outcomes, or quality of life, were included. Particular emphasis was placed on interventional studies, especially randomized controlled trials and controlled clinical studies. Observational and experimental studies were also considered; however, they were discussed separately within the narrative part of the review.

Studies were excluded if they focused exclusively on mineral antioxidants, were not related to endometriosis, or did not provide data relevant to the scope of this review. Due to the heterogeneity of the available studies in terms of design, population, intervention, and outcome measures, a selective approach was applied when preparing the clinical summary presented in Table 1. Therefore, the table should be interpreted as a structured overview of selected clinically relevant studies rather than a complete summary of all available evidence.

Some publications in Table 1 may report different outcomes derived from the same or overlapping clinical cohorts. When potential overlap was suspected, the findings were interpreted cautiously and not treated as fully independent evidence.

### 2.4. Study Selection Process

**Figure 1:**
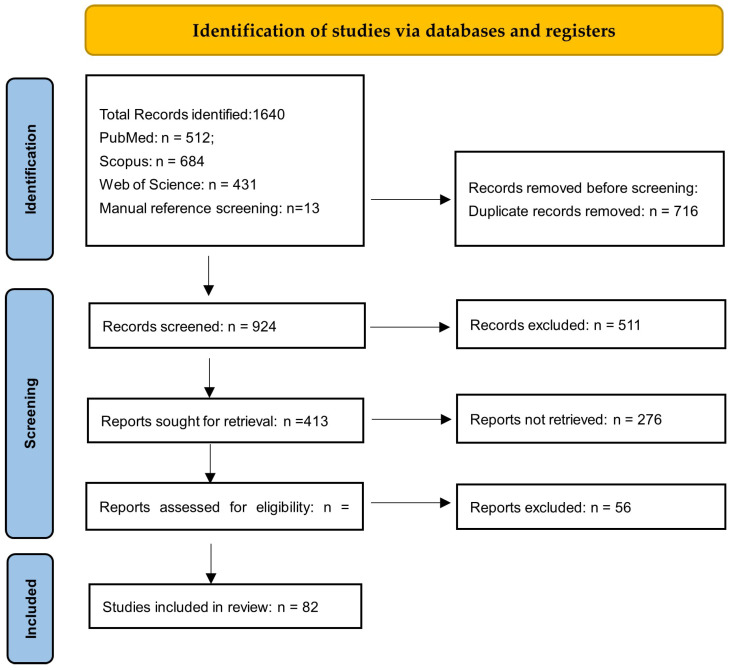
PRISMA 2020 flow diagram illustrating the study selection process.

All records identified through the database search were screened based on titles and abstracts. Articles considered potentially relevant were retrieved in full text and assessed for eligibility. The screening process was conducted independently by two reviewers. Any disagreements regarding study inclusion were resolved through discussion until consensus was reached. Reasons for exclusion at the full-text stage were recorded. The study selection process followed the principles of the PRISMA guidelines for transparent reporting of literature searches (Figure 1).

### 2.5. Data Extraction

Relevant information was extracted from all eligible studies and summarized in a standardized manner. For clinical studies, the extracted data included study design, sample size, characteristics of the study population, type and dosage of the intervention, duration of supplementation, and reported outcomes.

## 3. Overview of Included Studies

A total of 81 studies were included after the screening and eligibility assessment process (Figure 1). The material analyzed in this review consists of a mix of mechanistic studies, observational research, and clinical trials. Only a relatively small proportion of these were randomized or controlled clinical studies directly evaluating non-mineral antioxidant supplementation in women with endometriosis. The included studies vary considerably with regard to patient populations, disease stage, type and dose of supplementation, duration of intervention, and the outcomes assessed, such as pain intensity, oxidative stress markers, reproductive parameters, and quality of life, which limits direct comparison between studies. Because of this variability, the findings are presented as a narrative synthesis, with particular attention to clinically relevant observations.

## 4. Oxidative Stress in Endometriosis

Oxidative stress is a significant mechanism that perpetuates chronic inflammation and the progression of endometriosis. Excessive production of reactive oxygen species (ROS) has been demonstrated to promote immune dysregulation and enhanced growth and persistence of endometrium-like tissue in ectopic locations [28,29]. Clinical studies have repeatedly demonstrated elevated levels of oxidative stress markers, including lipid peroxidation products, and impaired total antioxidant capacity in serum, peritoneal fluid, and follicular fluid in women diagnosed with endometriosis. These findings substantiate the local and systemic character of redox dysfunction [30].

The findings of this study indicate that redox imbalance is not merely a secondary consequence of the disease, but rather, it may play an active role in its perpetuation and exacerbation, particularly in the manifestation of chronic pain symptoms. The physiological regulation of ROS (reactive oxygen species) is contingent upon the interplay of enzymatic and non-enzymatic antioxidant mechanisms. In women diagnosed with endometriosis, there is an observed alteration in the activity of key antioxidant enzymes. This finding is indicative of a redox response dysfunction and an increased tissue susceptibility to oxidative damage, as well as the activation of inflammatory pathways [31,32]. Given the established role of oxidative stress in the pathophysiology of endometriosis and its potential impact on the severity of clinical symptoms, it is reasonable to analyze interventions aimed at restoring redox balance. In this context, non-mineral antioxidant strategies that can complement standard treatment are of particular interest.

From a mechanistic perspective, the discussed supplements may affect different elements of the proposed redox-dependent network. Vitamins C and E act mainly as direct antioxidants. Melatonin is associated with mitochondrial function and inflammatory pathways. *N*-acetylcysteine supports glutathione synthesis and cellular redox balance, while coenzyme Q10 is involved in mitochondrial energy metabolism.

These observations suggest that different compounds may act on different aspects of the disease. However, it should be noted that most of these findings are derived from experimental and observational studies, and their direct clinical applicability remains limited.

## 5. Integrated Redox-Dependent Pathogenetic Model in Endometriosis

In addition to classical oxidative stress markers, emerging evidence suggests that endometriosis can be conceptualized as a self-sustaining, redox-driven inflammatory disorder, in which iron overload, mitochondrial dysfunction, inflammasome activation, and estrogen signaling form a complex interplay within a pathogenic network [9,33].

A central initiating factor appears to be iron-dependent on oxidative stress. Repeated cyclic bleeding within ectopic lesions leads to hemoglobin degradation and local iron accumulation in the peritoneal cavity. Excess ferrous iron (Fe^2+^) promotes Fenton reactions, generating highly reactive hydroxyl radicals and amplifying lipid peroxidation. This environment favors glutathione depletion and impaired glutathione peroxidase 4 (GPX4) activity, creating conditions conducive to ferroptosis. However, accumulating data suggest that endometriotic cells may develop partial resistance to ferroptosis cell death, allowing survival in a pro-oxidative milieu while surrounding tissues remain exposed to oxidative injury [34,35]. Concomitantly, the impairment of mitochondrial function and disruption of mitochondrial dynamics exacerbate the overproduction of ROS. Excessive mitochondrial fission (e.g., DRP1 activation) and reduced fusion signaling (MFN1/2, OPA1 imbalance) promote mitochondrial fragmentation and impaired oxidative phosphorylation efficiency. Fragmented mitochondria exhibit increased electron leakage and sustained superoxide generation, subsequently amplifying intracellular oxidative damage. This creates a vicious cycle in which ROS damages mitochondrial DNA and respiratory complexes, leading to persistent redox imbalance and metabolic reprogramming that favors lesion survival [36,37].

Excessive mitochondrial ROS serves as a potent activator of the NLRP3 inflammasome, particularly in peritoneal macrophages. ROS-dependent NLRP3 activation promotes caspase-1-mediated maturation of interleukin IL-1β and IL-18, amplifying local inflammatory signaling. This inflammasome-driven cytokine release contributes to neuroinflammation, peripheral sensitization, and chronic pelvic pain. Therefore, oxidative stress is not merely a consequence of inflammation but also a key initiator of innate immune activation [38].

**Figure 2:**
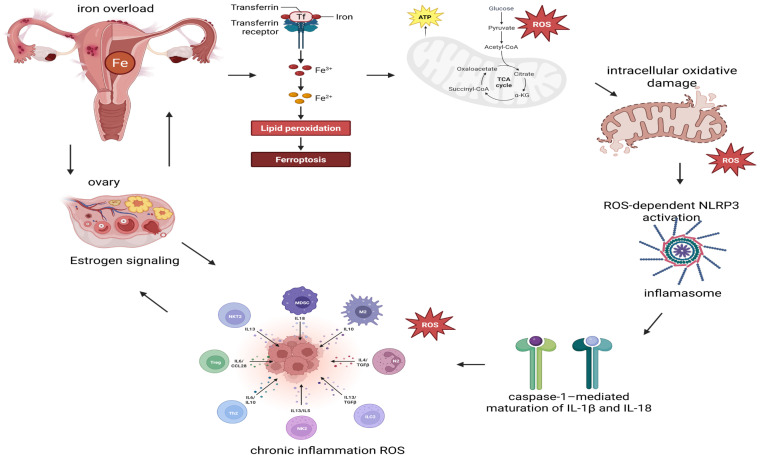
Integrated redox-dependent pathogenetic model in endometriosis. Created in BioRender. Szczuko, M. (2026). https://BioRender.com/fctn0b7. ATP—adenozyno-trifosforan; Fe—iron; IL—interleukin; NLRP3—protein containing NOD, LRR and pyrin 3 domains; ROS-reactive oxygen species.

Overlaying these mechanisms is a bidirectional estrogen–ROS crosstalk. Estrogen enhances mitochondrial respiration and can increase ROS generation, while oxidative stress upregulates aromatase expression within ectopic lesions, promoting local estrogen biosynthesis. This feed-forward loop sustains cellular proliferation, angiogenesis, and inflammatory activation. Estrogen signaling also modulates mitochondrial function and may indirectly influence ferroptosis susceptibility, further integrating hormonal and redox regulation (Figure 2) [39,40]. Collectively, these pathways form a self-amplifying pathogenic circuit:

- Iron accumulation → ROS overproduction → lipid peroxidation
- Mitochondrial fragmentation → sustained ROS generation
- ROS → NLRP3 inflammasome activation → chronic inflammation
- ROS ↔ Estrogen signaling → lesion persistence and proliferation

This consolidated model suggests that oxidative stress in endometriosis is not a single linear mechanism but rather a multidimensional network linking metabolic dysregulation, immune activation, and hormonal signaling [41,42,43]. Such complexity accounts for the disappointing outcomes of conventional antioxidant supplementation, which often produces minimal symptomatic improvement and fails to induce lesion regression. Targeted modulation of specific redox-sensitive pathways—such as ferroptosis susceptibility, mitochondrial dynamics, or inflammasome signaling—may represent more promising future therapeutic directions than nonspecific antioxidant strategies alone [43,44]. 

While this model provides a useful conceptual and hypothesis-generating framework, it is primarily based on experimental data and should be interpreted with caution in the context of clinical application. However, the direct translation of these mechanistic insights into consistent clinical outcomes remains limited.

## 6. Rationale for the Administration of Non-Mineral Antioxidant Supplements in Endometriosis

The presence of an oxidative-antioxidant imbalance in endometriosis suggests that the body’s inherent defense mechanisms may be inadequate in adequately reducing chronic oxidative stress. In this context, there is a growing interest in non-mineral antioxidants supplied through diet or supplements. These antioxidants have the potential to supplement physiological protective systems and modulate the local inflammatory-oxidative microenvironment of endometrial lesions [28,45]. Non-enzymatic antioxidants of exogenous origin, including vitamins A, C, and E, as well as selected compounds with antioxidant properties used in supplementation, have been shown to reduce lipid peroxidation and oxidative protein damage in conditions of persistent inflammation characteristic of endometriosis. These mechanisms are of particular importance in situations of prolonged exposure to ROS, when the natural antioxidant capacity of tissues is depleted However, the clinical relevance of these mechanisms remains uncertain and requires confirmation in well-designed clinical studies [46,47,48].

Increasing evidence suggests that mitochondrial dysfunction may contribute to elevated ROS production in endometriosis. Impaired mitochondrial metabolism and iron homeostasis disorders may further exacerbate oxidative stress. However, the significance of individual molecular pathways in the pathogenesis of the disease in humans remains unclear [26]. Despite the consistent evidence indicating heightened oxidative stress and diminished antioxidant capacity in endometriosis, the outcomes of clinical trials assessing the efficacy of antioxidant interventions remain equivocal. The ambiguity in the interpretation of the available data is attributable to differences in study designs, disease stage, patient characteristics, and supplementation protocols [26,47,48,49,50]. However, the observations provide a biological rationale for the further evaluation of non-mineral antioxidant strategies as complementary methods aimed at modulating the symptoms of endometriosis by restoring redox balance. A review of available randomized clinical trials evaluating the effect of selected non-mineral antioxidant supplements on clinical symptoms and markers of oxidative stress in women with endometriosis is summarized in Table 1. The ensuing subsections will address a selection of compounds, with an emphasis on the findings from clinical trials and their potential therapeutic implications.

### 6.1. Vitamins

Vitamin C (ascorbic acid) is a pivotal water-soluble antioxidant. It has been demonstrated to neutralize reactive oxygen species, including superoxide anion radical, hydrogen peroxide, and hydroxyl radical. These compounds play an important role in the pathogenesis of oxidative stress in endometriosis [51].

In addition to its free radical scavenging activity, vitamin C influences the regulation of the inflammatory response and redox homeostasis by inhibiting NF-κB activation and activating the Nrf2 pathway, leading to a reduction in the expression of pro-inflammatory cytokines and a strengthening of endogenous antioxidant mechanisms [28,52]. These mechanisms are of particular importance in the context of endometriosis, a condition marked by persistent inflammatory activation and elevated oxidative stress [53].

At the cellular level, vitamin C has been observed to reduce the adhesion and proliferation of endometrial cells and modulate the expression of angiogenic factors, including VEGF. However, the clinical significance of these observations remains to be elucidated [32,54]. A significant component of its mechanism involves its functional association with the body’s intrinsic antioxidant system, encompassing the glutathione cycle. Disruptions in the glutathione cycle have been extensively documented in women afflicted with endometriosis [49]. The most thoroughly documented clinically relevant mechanism is the synergistic interaction of vitamin C with vitamin E, which consists of the regeneration of the oxidized form of α-tocopherol and the reduction of lipid peroxidation. In randomized clinical trials, combined supplementation with vitamins C and E has been associated with a reduction in markers of oxidative stress and a moderate reduction in pain severity. However, the effectiveness of vitamin C monotherapy remains limited and dependent on the initial antioxidant status and form of supply [51,55,56].

Considering the evident functional interdependence, it is rational to delve deeper into the role of vitamin E as a pivotal lipophilic antioxidant in safeguarding against oxidative stress in endometriosis.

Vitamin E encompasses a group of tocopherols and tocotrienols, among which α-tocopherol plays a dominant biological role, interrupting the chain reactions of lipid peroxidation in cell membranes and lipoproteins. The antioxidant activity of the substance in question is maintained in the lipid environment, among other things, by virtue of its capacity for regeneration through vitamin C [57]. In the context of endometriosis, the importance of vitamin E is primarily related to its capacity to reduce lipid peroxidation and to modulate inflammatory processes in the microenvironment of disease foci. Observational studies have indicated that women with endometriosis often have reduced concentrations of vitamin E or lower dietary intake, which correlates with increased oxidative stress and disturbed antioxidant balance [53,55,56]. Reduced concentrations of vitamins E and C in follicular fluid may further contribute to increased lipid peroxidation and chronic inflammation within the ovary [58,59].

A review of the extant clinical studies reveals the existence of short-term, randomized, placebo-controlled trials in which vitamin E was supplemented exclusively in conjunction with vitamin C. The findings of these trials indicate a reduction in the severity of pelvic pain, painful menstruation, and dyspareunia, as well as an improvement in redox balance parameters, expressed as a decrease in lipid peroxidation markers and an increase in total antioxidant capacity [14,47]. However, it should be noted that supplementation does not lead to the regression of endometriotic lesions and should be regarded as a supportive intervention [13,14,60]. The efficacy of vitamin E monotherapy remains unconfirmed, and the long-term use of high doses necessitates caution due to the paucity of available safety data [47,55].

In contrast to vitamins C and E, which possess direct antioxidant properties, vitamin D exerts its effects on oxidative stress primarily indirectly, by modulating inflammatory and immune responses. It is a fat-soluble compound whose biological significance extends beyond the regulation of calcium and phosphate metabolism. Increasing evidence suggests that it plays a significant role in modulating the immune response and inflammatory processes. These mechanisms have drawn attention to the potential role of vitamin D in chronic inflammatory diseases, including endometriosis [61]. A compelling argument substantiates the biological role of vitamin D in endometriosis, primarily hinging on the presence of its receptor (VDR) and enzymes responsible for local activation and inactivation of the hormone (CYP27B1, CYP24A1) in both normal endometrium and ectopic foci of the disease. This argument is further bolstered by the presence of these enzymes in immune system cells present in their microenvironment [62]. These mechanisms are of particular importance in the context of persistent inflammatory activation, immune response disorders, and abnormal angiogenesis, which are characteristic of the pathogenesis of the disease. This phenomenon enables local paracrine and autocrine action, thereby affecting macrophage activity and the balance of Th1/Th17 lymphocyte responses, which are pivotal to maintaining chronic inflammation [63]. It is important to note that vitamin D does not act as a classic antioxidant, neutralizing free radicals. However, it can indirectly influence the redox balance by inhibiting inflammatory pathways that promote excessive production of reactive oxygen species [64]. A moderate analgesic effect of supplementation has been suggested by observational and interventional data, particularly in patients with baseline vitamin D deficiency. However, the results of studies have been inconsistent, and the effect on markers of oxidative stress has been limited [64].

Consequently, vitamin D should be regarded as an indirect modulator of inflammatory and immune processes rather than a conventional antioxidant, and its clinical utilization appears most substantiated in patients with substantiated deficiency. The subsequent subsection will address vitamin A and its derivatives, the role of which in endometriosis has been primarily analyzed through observational studies.

Vitamin A and its biologically active derivatives, known as retinoids, are non-mineral, fat-soluble compounds that play a crucial role in regulating various physiological processes, including cell differentiation, immune response, and inflammatory processes [57,65]. Although vitamin A is not a classic antioxidant that directly neutralizes reactive oxygen species, retinoids can modulate oxidative stress pathways indirectly by regulating the expression of genes involved in inflammation, angiogenesis, and estrogen signaling. These pathways are important for the pathophysiology of endometriosis [65,66].

The biological activity of retinoids is predominantly facilitated by retinoic acid nuclear receptors (RAR) and retinoid X receptors (RXR), which modulate the expression of genes implicated in immune response, cell proliferation, and angiogenesis. The capacity of RXR to form heterodimers with other nuclear receptors, including the vitamin D receptor and the PPAR receptors, suggests the possibility of interactions between retinoid signaling and mechanisms regulating inflammation and redox balance [67,68]. Despite the presence of biologically coherent premises, there are currently no randomized clinical trials evaluating the effect of vitamin A or retinoid supplementation on symptoms, oxidative stress markers, or reproductive parameters in women with endometriosis. Consequently, their function within the framework of targeted antioxidant supplementation remains negligible and is primarily constrained to mechanistic hypotheses necessitating additional clinical validation [54,57,66].

### 6.2. Melatonin

Considering the limited clinical evidence available for vitamin A, melatonin currently appears to be the non-mineral antioxidant with the most extensively investigated potential in endometriosis. Melatonin is an endogenous indoleamine hormone synthesized primarily in the pineal gland, and it is well-known for regulating the circadian rhythm. In addition to this function, melatonin exhibits well-documented antioxidant, anti-inflammatory, and immunomodulatory properties [69,70]. These features have led to growing interest in its potential use as a supportive intervention in endometriosis, a condition in which oxidative stress and chronic inflammatory activation contribute to disease pathophysiology and pain development [71].

The antioxidant activity of melatonin is mediated through both direct and indirect mechanisms. It can directly neutralize reactive oxygen and nitrogen species, while also enhancing the activity of endogenous antioxidant enzymes and inhibiting pro-oxidative pathways. In addition, melatonin has been shown to influence mitochondrial function by stabilizing the respiratory chain and limiting mitochondrial ROS production, which may be particularly relevant in the context of endometriosis-associated oxidative imbalance [70,71,72]. Experimental studies further suggest that melatonin may exert anti-proliferative effects on endometrial cells and may modulate angiogenic and inflammatory signaling, including the downregulation of selected inflammatory mediators [62,72,73].

The most consistent clinical observations relate to the potential effects of melatonin on pain symptoms. Several clinical studies, including randomized trials and meta-analyses, have reported reductions in pain intensity and improvements in quality of life, although these effects appear to be independent of sleep-related benefits [21,23]. However, the current strength of clinical evidence remains moderate due to several factors. First, the limited number of randomized trials conducted thus far has hindered the ability to draw definitive conclusions. Second, the heterogeneity of supplementation protocols has led to a lack of standardization of endpoints, which is crucial for the reliability of study results. Despite the promising results of recent studies [24], the field still faces significant challenges in generating high-quality evidence.

Taken together, melatonin may represent one of the more biologically plausible non-mineral antioxidant interventions investigated in endometriosis to date. However, given the current limitations of clinical evidence, its potential role should be considered supportive, and further well-designed clinical trials are needed to better define its therapeutic value.

### 6.3. N-acetylcysteine

*N*-acetylcysteine (NAC) is a non-mineral thiol compound that serves as a precursor of cysteine, an amino acid required for the synthesis of glutathione, one of the key components of the endogenous antioxidant defense system. Through this mechanism, NAC supplementation may increase intracellular glutathione levels and contribute to the restoration of redox balance [74]. In addition to its indirect effects on the glutathione system, NAC also exhibits direct antioxidant activity and has been shown to modulate inflammatory signaling pathways, including NF-κB and COX-2, which are known to be upregulated in endometriotic lesions [52,53,75].

The available clinical studies, although limited in number and methodologically heterogeneous, suggest that NAC supplementation may provide some benefits in women with endometriosis. Reported effects include reductions in pain symptoms and, in some cases, decreases in the size of endometriotic lesions. However, the overall strength of clinical evidence remains limited. The number of randomized controlled trials is small, and existing studies differ considerably in terms of dosing regimens, treatment duration, and outcome measures. These factors make comparisons between studies difficult and restrict the strength of conclusions that can be drawn [75]. Taken together, NAC represents a biologically plausible antioxidant strategy that may have potential relevance in endometriosis. However, current clinical evidence is insufficient to support firm therapeutic recommendations, and further well-designed randomized trials are needed to clarify its role as an adjunctive intervention (Table 1).

Strategies aimed at improving redox balance may also involve compounds that directly influence mitochondrial function. Among these, coenzyme Q10 has attracted particular attention.

### 6.4. Coenzyme Q10

Coenzyme Q10 (ubiquinone) is an endogenous, fat-soluble compound that plays a pivotal role in the mitochondrial respiratory chain, where it is involved in electron transport and cellular energy production. In addition to its bioenergetic functions, it has antioxidant properties, which limit lipid peroxidation and protect mitochondrial structures from oxidative damage [76].

Increasing evidence suggests that mitochondrial dysfunction plays a substantial role in the development of oxidative stress in endometriosis, contributing to chronic inflammation, energy metabolism disorders, and pain. Preliminary clinical investigations indicate that coenzyme Q10 may enhance mitochondrial function, curtail excessive production of reactive oxygen species, and modulate inflammatory pathways associated with NF-κB activation. However, these observations are primarily derived from experimental models [77].

The clinical data on the use of coenzyme Q10 in women with endometriosis are limited and largely indirect, often involving populations of patients with infertility or chronic pelvic pain. Consequently, the current strength of evidence remains low to moderate, and coenzyme Q10 should be regarded as a component of adjunctive therapy, necessitating further substantiation through randomized clinical trials [76,77,78,79].

Table 1 presents the characteristics of randomized clinical trials evaluating the effect of non-mineral antioxidant supplements on clinical symptoms and markers of oxidative stress in women with endometriosis. The included studies differed in terms of design, sample size, type and dose of supplement, duration of intervention, and endpoints assessed. Most studies focused on the severity of pain, while some also encompassed the evaluation of oxidative stress markers, reproductive parameters, or quality of life [80,81,82]. The results presented herein indicate significant discrepancies in the quality and consistency of the evidence, which necessitates further interpretation. These differences substantially limit the comparability of the studies and should be taken into account when interpreting the clinical relevance of the findings.

## 7. Non-Mineral Antioxidant Supplements and Oxidative Stress in Endometriosis: Clinical Implications

A summary of the clinical trials presented in Table 1 indicates that the observed outcomes of non-mineral antioxidant supplementation in endometriosis are heterogeneous and may depend on the specific intervention, duration of supplementation, and characteristics of the study population. Overall, Table 1 shows considerable variability in study design, outcomes, and quality of evidence, which makes direct comparison between interventions difficult. The evidence includes randomized trials, observational studies, and mechanistic data, which differ in strength and direct clinical relevance. The available data indicate that strategies aimed at modulating oxidative stress may have some impact on disease symptoms; however, the strength and consistency of clinical evidence vary between individual compounds. Table 1 should be understood as a structured summary of selected clinically relevant studies rather than a comprehensive overview of all available evidence. Importantly, many of the proposed mechanisms are derived from preclinical research and do not directly translate into clinical effectiveness.

Among the interventions discussed, melatonin appears to have the most consistent clinical support, with randomized controlled trials indicating a moderate reduction in pain and improvement in quality of life. Its proposed mechanisms include effects on redox balance, mitochondrial function, and inflammatory pathways. At the same time, the limited number of studies and variability in supplementation protocols highlight the need for further well-designed clinical trials.

For vitamins C and E, some studies suggest moderate, mostly short-term benefits, particularly when used in combination. Reported effects include reductions in oxidative stress markers and modest improvements in pain symptoms. However, these findings are not consistent across studies, and the lack of evidence for monotherapy and lesion regression limits the possibility of drawing firm clinical conclusions. Therefore, these vitamins may be considered supportive interventions in selected cases.

*N*-acetylcysteine appears biologically promising, given its role in modulating the glutathione system, mitochondrial function, and inflammatory pathways. Some clinical studies suggest potential benefits in terms of pain reduction and disease progression; however, the available evidence remains limited and heterogeneous, and is largely based on non-randomized or complex intervention studies. As such, its clinical relevance requires further confirmation.

Vitamin D may indirectly influence redox balance through its effects on immune and inflammatory processes. Clinical data suggest a limited analgesic effect, particularly in patients with baseline deficiency, although results remain inconsistent. For vitamin A, despite a plausible biological rationale, there is currently insufficient clinical evidence to assess its therapeutic relevance in endometriosis. Therefore, its role remains largely theoretical at the current stage of research. Similarly, coenzyme Q10 represents a potentially interesting approach due to its mitochondrial effects, but clinical data are limited and do not allow for clear conclusions.

Overall, non-mineral antioxidant supplements should not be considered a homogeneous group of interventions. Rather, they represent a diverse category of compounds with different mechanisms of action and varying levels of clinical evidence. Their potential use in endometriosis should be considered on an individual basis and primarily as an adjunct to standard treatment.

When comparing the discussed interventions, differences in the strength of evidence and potential clinical relevance become apparent. Overall, melatonin shows the most consistent support from randomized clinical trials, while vitamins C and E demonstrate moderate but less consistent effects.

In contrast, *N*-acetylcysteine and coenzyme Q10 exhibit strong biological plausibility but remain supported by limited and heterogeneous clinical evidence. Therefore, although several compounds appear promising, the current data do not allow for clear conclusions regarding their comparative effectiveness, and further well-designed clinical studies are required.

One possible explanation for the limited clinical efficacy of antioxidant supplementation is that oxidative stress in endometriosis may act more as an amplifier of ongoing pathological processes rather than a primary driver. In addition, redox processes may be locally regulated, and systemic antioxidant therapy may not adequately reflect these local conditions. Reactive oxygen species also play both damaging and regulatory roles, and their non-selective reduction may affect normal cellular signaling without clearly improving disease-related pathways. Furthermore, the involvement of multiple overlapping mechanisms may limit the effectiveness of interventions targeting a single pathway.

## 8. Strengths and Limitations

A significant strength of this study is its comprehensive approach to the role of oxidative stress in the pathophysiology of endometriosis. The study covers both molecular mechanisms and available clinical data on non-mineral supplements with potential antioxidant activity. The analysis incorporates compounds that affect various levels of disease processes, ranging from the regulation of redox balance and modulation of the inflammatory response to the impact on mitochondrial function. This comprehensive approach enables a multidimensional assessment of their potential clinical significance. A critical methodological component pertains to the consistent differentiation between preclinical, observational, and interventional study data, in addition to the judicious evaluation of the strength of the extant evidence, with consideration for its quality and limitations.

The limitations of this study stem primarily from the nature of the available literature. For a considerable number of the supplements under discussion, the number of randomized clinical trials remains limited, and the existing studies are characterized by significant heterogeneity in terms of study populations, doses, duration of intervention, and endpoints utilized. For certain compounds, notably vitamin A and its derivatives, the extant evidence is predominantly mechanistic or observational, thereby further constricting the capacity to draw conclusions regarding the clinical significance of supplementation.

A further limitation is the absence of uniform, universally accepted markers of oxidative stress in clinical trials. This absence makes it difficult to directly compare results between different interventions. Due to the substantial heterogeneity in study designs, interventions, and endpoints assessed, it was not methodologically feasible to perform a quantitative meta-analysis. Consequently, the conclusions presented should be regarded as a synthetic, qualitative assessment of the current state of knowledge, rather than a formal systematic review, requiring further verification in well-designed clinical trials. Another limitation of this review is the lack of a standardized framework, such as GRADE (Grading of Recommendations Assessment, Development and Evaluation), to formally assess the quality of evidence and the strength of recommendations. This reflects the narrative design of the study and the heterogeneity of the available data.

Other non-mineral antioxidants, including resveratrol, hydroxytyrosol, and omega-3 fatty acids, have also been explored in endometriosis. These compounds were not included in the present review due to the defined scope, but they may represent potential directions for future research.

## 9. Future Directions

Future studies should further clarify the role of oxidative stress in endometriosis and its relevance to clinical outcomes. In particular, it remains unclear to what extent oxidative stress represents a primary driver of the disease or a secondary phenomenon. Better characterization of patient populations and disease heterogeneity may help to identify subgroups more likely to benefit from antioxidant-based approaches. At the same time, more targeted strategies addressing specific components of redox regulation should be explored. Additional well-designed clinical studies are required to better define their potential therapeutic role.

## 10. Conclusions

Oxidative stress appears to play an important role in the pathophysiology of endometriosis, particularly in relation to chronic inflammation, mitochondrial dysfunction, and pain-related mechanisms. This provides a biological rationale for considering antioxidant-based strategies as a potential supportive approach. However, the current clinical evidence on non-mineral antioxidant supplementation remains limited and heterogeneous. Among the compounds discussed, melatonin shows the most consistent, although still moderate, level of clinical support, while *N*-acetylcysteine appears promising but is supported by relatively few and methodologically diverse studies. For vitamins C, E, and D, as well as coenzyme Q10, the available data are inconsistent or insufficient to support clear clinical conclusions. In the case of vitamin A, the lack of clinical studies precludes assessment of its therapeutic relevance. Overall, non-mineral antioxidant supplementation should not be considered a standalone treatment for endometriosis. These interventions may have a role as supportive strategies in selected patients, but their clinical value remains uncertain. Further well-designed randomized clinical trials are needed to better define their effectiveness and place in therapeutic management.

## Figures and Tables

**Table 1:** Clinical trials of non-mineral antioxidant supplementation in endometriosis. This table represents a selective synthesis of clinically relevant interventional studies and should not be interpreted as an exhaustive summary of all available evidence. Due to substantial heterogeneity in study design, populations, interventions, and outcome measures, a focused approach was adopted to improve clinical interpretability.

Supplement	Study Design/Population (n)	Dose and Duration	Main Outcomes	Key Limitations	Level of Evidence	References
Vitamin C + E	RCT; n = 46 vs. 13 placebo.	Vit. C 1000 mg/d + Vit. E 1200 IU/d for 8 weeks	↓ pelvic pain ↓ dysmenorrhea	small sample size, short duration	Moderate	[11]
Vitamin C + E	RCT; n = 30 vs. 29 placebo	Vit. C 343 mg/d + Vit. E 84 mg/d for 8 weeks	↓ lipid peroxidation ↓ oxidative stress no fertility effect	heterogeneous endpoints, small sample size, short duration	Moderate	[12]
Vitamin C + E	RCT; n = 30 vs. 30 placebo	Vit. C 1000 mg/d + Vit. E 800 IU/d for 12 weeks	↓ oxidative stress markers ↑ antioxidant capacity	limited endpoints, small sample size, short duration	Moderate	[13]
Vitamin C + E	RCT; n = 30 vs. 30 placebo	Vit. C 1000 mg/d + Vit. E 1200 IU/d for 8 weeks	↓ pelvic pain, ↓ dysmenorrhea ↓ dyspareunia	heterogeneous endpoints, small sample size, short duration	Moderate	[14]
Vitamin C	Controlled study; 160 cases/120 placebo + 150 controls	Vit. C 1000 mg/d for 6 weeks	↑ follicular fluid ↑ serum vitamin C levels	no clinical endpoints short duration	Low	[15]
Vitamin C + E +A	RCT; n = 37 vs. 35 placebo	Vit. C 500 mg/d + Vit. E 20 mg/d + Vit. A 1050 ug/d for 4 months	↑ SOD ↑ GPx ↓ MDA ↓ LPH	biomarker-focused outcomes, small sample size	Moderate	[16]
Vitamin D_3_	RCT; n = 19 vs. 20 placebo	Vit. D_3_ 50,000 IU weekly for 12 weeks	no effect on pain	small sample size	Low	[17]
Vitamin D_3_	RCT; n = 35 vs. 35 placebo	Vit. D_3_ 2000 IU/d for 24 weeks	no effect on pain, analgesic use, quality of life	negative findings, small sample size	Moderate	[18]
Vitamin D_3_	RCT; n = 30 vs. 30 placebo	50,000 IU vit. D_3_ every 2 weeks for 12 weeks	↓ pelvic pain, ↓ total-/HDL-cholesterol ratio, ↓ hs-CRP ↓ TAC	small sample size	Moderate	[19]
Vitamin D_3_	RCT; n = 17 vs. 17 placebo	50,000 IU vit. D_3_ weekly for 12–14 weeks	↓ the ratio of active/total form of β-catenin protein expression	small sample size	Low	[20]
Melatonin	RCT; n = 20 vs. 20 placebo	Melatonin 10 mg nightly for 8 weeks	↓ pain (39.8%), ↓ dysmenorrhea (38%), ↑ sleep quality	small sample size	Moderate	[21]
Melatonin	RCT; n = 20 vs. 20 placebo	Melatonin 20 mg nightly for 2 months	no significant pain reduction	small sample size	Moderate	[22]
Melatonin	RCT; n = 40 vs. 40 placebo	Melatonin 5 mg nightly for 2 months	moderate pain reduction ↑ sleep quality	limited endpoints	Moderate	[23]
Melatonin	RCT; n = 49 vs. 49 placebo	Melatonin 10 mg nightly for 8 weeks	↓ dysmenorrhea	limited endpoints	Moderate	[24]
*N*-acetylcysteine (NAC)	Prospective clinical studies, n = 47 vs. 45 placebo	NAC 600 mg 3× daily for 3 months	↓ pain symptoms, ↓ endometrioma size, ↓ need for surgery	lack of RCTs, heterogeneous protocols	Low	[25]
Coenzyme Q10	Limited clinical evidence, no RCT	variable dosing regimens	↑ mitochondrial function ↓ oxidative stress	No direct RCTs in endometriosis	Low	[26,27]

SOD—superoxide dismutase, GPx—glutathione peroxidase, LPH—lipid hydroperoxide; MDA—malondialdehyde. ↑—increase; ↓—decrease.

## Data Availability

The original contributions presented in this study are included in the article. Further inquiries can be directed to the corresponding authors.

## Abbreviations

The following abbreviations are used in this manuscript:

GPX4	Glutathione peroxidase 4
NAC	*N*-acetylcysteine
ROS	Reactive oxygen species
RAR	Retinoic acid nuclear receptors
SOD	Superoxide dismutase
